# Valuing lives: Allocating scarce medical resources during a public health emergency and the Americans with Disabilities Act (perspective)

**DOI:** 10.1371/currents.RRN1271

**Published:** 2011-09-21

**Authors:** Leslie Wolf, Wendy Hensel

**Affiliations:** Professor of Law, Georgia State University College of Law, Atlanta, GA

## Abstract

Public health emergencies from natural disasters, infection, and man-made threats can present ethically or legally challenging questions about who will receive scarce resources. Federal and state governments have offered little guidance on how to prioritize distribution of limited resources. Several allocation proposals have appeared in the medical literature, but components of the proposed approaches violate federal antidiscrimination laws and ethical principles about fair treatment. Further planning efforts are needed to develop practical allocation guidelines that comport with antidiscrimination laws and the moral commitment to equal access reflected in those laws.

**Word count**: 1178

**Keywords**:  public health emergency, allocation scarce resources, disability law

**Tables and figures**:  1   

      The September 11 bombings and the anthrax scare prompted federal, state, and local authorities in the United States to engage in advance planning efforts for widespread public health emergencies.  Hurricane Katrina highlighted important gaps in government responses to the disaster.[1]  For our purposes, one is particularly relevant.  As illustrated most dramatically in the case of Anna Pou, the New Orleans physician who was accused of murdering patients, medical personnel faced challenging decisions about which patients would receive what kind of care under extreme conditions, including lack of electrical power and accurate information about relief efforts, and severe shortages of personnel and medical equipment.[2]  Importantly, they were asked to make those decisions with essentially no outside guidance.

      In the absence of government direction, several independent groups have developed plans for allocating scarce resources during a public health emergency, focusing particularly on ventilators and other critical care.[3] [4] [5] [6] [7] These efforts are intended to provide specific, practical guidelines that could be implemented in a crisis.  The underlying goal is to maximize the number of people who survive to hospital discharge by incorporating criteria that distinguishes between patients.  Most protocols rely on the Sequential Organ Failure Assessment (SOFA) as a key, objective measurement for evaluating patient health status.[3] [4] [5] [6]  Patients who fall below a certain SOFA score are not eligible for ventilator support in times of scarcity.  Patients may also be excluded from ventilation for specific health conditions, which vary between protocols. 

      Some of these proposed exclusions run afoul of federal antidiscrimination laws, including the Rehabilitation Act and the Americans with Disabilities Act (ADA).[8] [9] These laws broadly preclude discrimination against people with disabilities, which is defined as mental or physical impairment that “substantially limits” at least one “major life activity,” on the basis of their impairments, and clearly apply to provision of medical care in almost all settings. These laws seek to provide people with disabilities with equal access and opportunities and require reasonable accommodation to meet these goals, unless such accommodation would result in a fundamental alteration of the service.  Services may be denied on the basis of disability when the person poses a direct threat to safety and well-being of others (e.g., because of infectious disease), but such a determination requires an individualized assessment and must be based on objective knowledge.  The protections afforded by these laws would apply equally to those who become disabled as a result of the public health emergency and those whose disability preceded the emergency.

      It is essential that those developing allocation protocols understand that, as we describe in more detail elsewhere, there are several ways in which the protocols may violate federal antidiscrimination laws.[9]  First, there are categorical exclusions based on specific disabling conditions, such as “severe cognitive impairment.”  Severe cognitive impairment is not defined, and, thus, could apply to a wide variety of conditions from Down’s syndrome, to schizophrenia, to Alzheimer’s.  Such categorical exclusions are not based on the patient’s likelihood of survival, but instead reflect a qualitative assessment of the lives that deserve to be saved.  Relatedly, some protocols include quality of life assessments as an explicit criterion to exclude some patients from care.  Both approaches are problematic.  Quality of life considerations are not “neutral,” even when cast in medical terms. There is significant evidence that an assessment of quality of a life by *any* non-disabled individual will systematically undervalue the quality of life with disabilities, particular mental disabilities.[10]  Such exclusions are precisely the type of subjective decision-making that federal antidiscrimination laws sought to preclude.

      Other criteria appear facially neutral and medically based, such as the duration of the patient’s need for care and that depend on an evaluation of medical effectiveness, but may, in some cases, violate antidiscrimination laws.  The duration of care criterion may have a disparate impact on people with disabilities if, for example, their disability necessitates additional time on the ventilator to achieve the same effect as for the non-disabled.  Although case law does not provide a clear answer, whether this criterion passes muster will likely depend on whether the limitation provides an equal or meaningful opportunity to obtain the same benefit or results as offered to non-disabled individuals.  People with disabilities who require short term ventilation because of influenza may plausibly argue that they are not seeking additional substantive benefits, but instead a reasonable modification --- an extension of time – to facilitate meaningful access to the same benefit – a ventilator.  However, the strength of this argument weakens as the need for ventilation lengthens.  Courts have typically interpreted the antidiscrimination laws to preserve medical judgment pertaining to treatment.  Thus, the medical effectiveness criterion would seem consistent with federal antidiscrimination laws to the extent that this evaluation is limited to the most basic question of whether a particular patient will survive or receive a physiological benefit from implementation of the scarce resource.  For example, although individuals with severe disabilities may be more likely to receive low SOFA scores that exclude them from receiving ventilators, such evaluation is objectively based on organ function, rather than on the disability per se.  On the other hand, if “effectiveness” or “benefit” is defined in such a way as to prefer individuals with no significant preexisting medical disorders, it may once again run afoul of the ADA and the Rehabilitation Act. Table 1 summarizes this analysis of proposed allocation factors in terms of consistency with federal antidiscrimination laws.  As discussed, factors are more likely to be consistent with the laws to the extent that they focus on documented medical effects of a particular medical condition (e.g., lung function) on response to treatment, rather than on assumptions about the effect of the disability either on treatment or on quality of life.

      Some might respond to our critique that the problem lies not with the proposed allocation protocols, but with the federal antidiscrimination laws; that the requirements of the laws must give way in a state of crisis.  However, that position is at odds with the federal government’s demonstrated commitment to addressing the needs of people with disabilities during emergencies.[1]  Although federal law already prohibited discrimination in emergency responses, in 2004, President Bush ordered federal agencies to take the needs of people with disabilities into account when developing emergency preparedness plans.  A 2006 law established a Disability Coordinator to ensure government preparedness plans and disaster relief efforts attended to the needs of people with disabilities.  There are no provisions for waiving requirements of antidiscrimination laws during an emergency, nor should there be.  We must honor our commitment to all our citizens in time of crisis.

      Our analysis suggests the need for additional work on emergency preparedness.  Practical, implementable allocation protocols are needed, but they must also be consistent with existing antidiscrimination laws.  The laws do not preclude triage of patients necessitated by a public health emergency, but do preclude disadvantaging people with disabilities because of their disabilities.  Because, ultimately, allocation protocols for public health emergencies will only succeed if the public accepts them as reasonable and fair, we join in the call for public engagement on this issue,[11] which must include voices from the disability community to avoid the biases described here.   

**Table 1:  Evaluation of allocation principles**

**Table:** 

**Consistent with antidiscrimination laws**	**Inconsistent with antidiscrimination laws**
Medical effectiveness (strictly defined and evidence based)	Medical effectiveness (broadly defined and not-evidence based)
Duration of care (evidence based)	Duration of care (not evidence based)
Categorical exclusions (evidence-based and focused on short term prognosis)	Categorical exclusions (not evidence-based and focused on quality of life)
	Quality of life assessments

**Acknowledgments**

The authors would like to thank Paul Lombardo for his thoughtful comments reviewing this work.  The complete legal analysis on which this paper is based was published in Hensel WF, Wolf LE (2011) Playing God:  The Legality of Plans Restricting People with Disabilities from Scarce Resources in Public Health Emergencies.  Florida Law Review 63:  719-770.

**Funding**

The research for this paper was supported, in part, through internal grants at the Georgia State University College of Law.

**Competing interests**

The authors have declared that no competing interests exist.
